# Nontyphoidal *Salmonella* and Their Antimicrobial Susceptibility among Diarrheic Patients Attending Private Hospitals in Addis Ababa, Ethiopia

**DOI:** 10.1155/2021/6177741

**Published:** 2021-09-18

**Authors:** Ruhama Kebede, Haile Alemayehu, Girmay Medhin, Tadesse Eguale

**Affiliations:** Aklilu Lemma Institute of Pathobiology, Addis Ababa University, P.O. Box 1176, Addis Ababa, Ethiopia

## Abstract

Nontyphoidal *Salmonella* (NTS) is one of the major causes of bacterial foodborne infection. It is mainly manifested by self-limiting gastroenteritis in healthy individuals but can also cause severe complications including blood stream infection and mortality. The emergence of multidrug-resistant strains of *Salmonella* is becoming a global public health concern. This study is aimed at estimating the prevalence of *Salmonella*, identifying serotypes involved, and investigating antimicrobial susceptibility of the isolates among diarrheic patients attending private hospitals in Addis Ababa. We collected a total of 298 stool samples from diarrheic patients attending five private hospitals in Addis Ababa and isolated *Salmonella* according to standard microbiological techniques; the isolates were serotyped using slide agglutination and microplate agglutination techniques. Antimicrobial susceptibility test of the isolates was carried out using Kirby-Bauer disc diffusion assay according to Clinical Laboratory Standards Institute guidelines. Fourteen stool samples (4.7%) were positive for *Salmonella*, and *Salmonella* Kiambu was the most dominant serovar (*n* = 7, 50%) followed by *S*. Saintpaul (*n* = 4, 28.6%) and *S.* Haifa (*n* = 2, 14.3%). Three (21.4%) of the isolates were resistant to sulfisoxazole and tetracycline each and 2 (14.3%) to ampicillin. Resistance to two antimicrobials was detected only in 2 (14.3%) of the isolates, and none of the isolates were resistant to more than two antimicrobials. In conclusion, the current study showed low prevalence of NTS in diarrheic patients attending private hospitals in Addis Ababa. Although multidrug resistance to several antimicrobials was not detected in the isolates, prudent use of antimicrobials is recommended to guaranty the long-term use of the available antimicrobials.

## 1. Introduction

Infection with NTS causes significant morbidity and mortality in humans and animals globally. Infection with NTS causes symptoms that can range from mild self-limiting gastroenteritis to severe invasive septicemia and can occasionally be fatal. Most of the infections caused by nontyphoidal *Salmonella* serovars are characterized by self-limiting acute gastroenteritis and diarrhea [1–3]. In addition to illness and death inflicted by NTS serovars, it was estimated to cause 4 million disability adjusted life years (DALYS) accounting for 22.2% of DALYS due to diarrhea causing agents that leads to huge economic loss [4]. In human, NTS infection usually causes foodborne outbreaks [3]. The common sources of infections are various animal products such as poultry, dairy, and pork [5]. *Salmonella* can also be transmitted from person to person, domestic animals like dogs, cats, and rodents, and consumption of contaminated products like sprouts, tomatoes, fruits, peanuts, and spinaches [5–7].

For decades, antimicrobials have reduced morbidity and mortality due to various bacterial infections worldwide. However, easy access to antimicrobials without prescription and extensive misuse both in public health and animal health sectors has led to emergence and spread of resistant strains of pathogenic bacteria [8, 9]. The emergence and rapid spread of antimicrobial resistance (AMR) in *Salmonella* has become a worldwide concern. In addition to resistance to first-line antimicrobials like third-generation cephalosporins and fluoroquinolones, plasmid borne resistance to colistin (the last resort antimicrobial for treatment of multidrug gram negative bacterial infections) mediated by different variants of *mcr* gene has been reported in *Salmonella* isolates from different countries [10]. An increasing number of treatment failures are often linked to multidrug resistant (MDR) *Salmonella* strains [11]. MDR *Salmonella* isolates have been associated with high risk of invasive infection, an increased risk of death, and prolonged illness as compared to infections caused by susceptible strains [12].

In Ethiopia, there are studies that indicated occurrence of *Salmonella* and high rate of antimicrobial resistance in both public health and veterinary sectors. For example, a study that investigated diarrheic patients attending health care facility in Gondar reported that 1.2% (4/372) of the stool samples were positive for *Salmonella* [13]. The isolates detected in this study were resistant to tetracycline (100%), amoxicillin (100%), and ampicillin (75%). Other study conducted in Nekemte referral hospital reported *Salmonella* in 2.1% (9/422) of the diarrheic study participants, and all of the isolates were resistant to amoxicillin [14]. In a study conducted in Southern Ethiopia, *Salmonella* was detected in 1.0% (2/204) of diarrheic patients and both isolates were resistant to ampicillin and gentamicin [15].

Most of the previous studies in Ethiopia were conducted on patients attending public health facilities, and little data is available on prevalence and antimicrobial susceptibility of *Salmonella* isolates from patients attending private health facilities. Due to difference in income, educational status and exposure to different predisposing factors, rate of *Salmonella* infection and antimicrobial susceptibility status of isolates might be different in people attending private health facilities compared to those attending government-owned health facilities. Estimating the prevalence of *Salmonella* among diarrheic patients and establishing antimicrobial susceptibility status of isolates combined with investigation of susceptibility of isolates to antimicrobials are of paramount importance to advice clinicians on appropriate management of *Salmonella* infection. Results from such study will also strengthen epidemiological knowledge of the disease that could help policy makers while planning interventions for the at-risk populations.

The objectives of this study were therefore (a) to estimate the prevalence of *Salmonella* among diarrheic patients attending private hospitals in Addis Ababa and to identify serotypes involved, (b) to investigate factors that can potentially be associated with *Salmonella* positivity, and (c) to assess antimicrobial susceptibility of *Salmonella* isolates.

## 2. Materials and Methods

### 2.1. Study Area, Context of the Study, and Study Design

The current study was conducted in Addis Ababa, the capital city of Ethiopia. The city lies at an elevation of 2,355 meters above sea level (m.a.s.l) and located at 9°1′48^″^ North and 38°44′24^″^ East, and it has a subtropical highland climate, with average annual temperature of 16.3°C and 1089 mm annual rainfall. Administratively, Addis Ababa was divided into 10 subcities, namely, Addis Ketema, Akaki-Kality, Arada, Bole, Gulele, Kirkos, Kolfe-Keranyo, Lideta, Nifas Silk-Lafto, and Yeka, when this study was conducted. According to World Population Review, population of Addis Ababa was estimated to be 4,591,983 in 2019 with average population density of 5,165 individuals per square kilometer [16].

Residents of Addis Ababa receive health services both from health institutions owned by the government and by private sectors. According to Addis Ababa City Administration Food, Medicine and Health Care Administration and Control Authority, there were more than 22 higher private hospitals in Addis Ababa in 2018 (personal communication). Although seven private hospitals were randomly selected, only five hospitals located in 4 of the 10 subcities in Addis Ababa agreed to participate in the study. Hence, diarrheic patients were recruited from these five private hospitals, namely, Bethezatha located in Kirkos subcity, Ethio Tebib in Addis Ketema subcity, iCMC and Migbaresenay Hospitals located in Yeka subcity, and Teklehaimanot Hospital located in Arada subcity. A cross-sectional study design was used to estimate prevalence of *Salmonella* in diarrheic patients by examining human stool samples and to determine antimicrobial susceptibility of the isolates.

### 2.2. Stool Sample Collection and Interviewing of Study Participants

Stool sample collection and patient interviews were conducted from September 2018 to June 2019. Two hundred and ninety-eight stool samples were collected from diarrheic patients attending five private hospitals during the study period. Screw-caped clean plastic containers were used for the collection of stool samples. The samples were transported to Microbiology Laboratory of Aklilu Lemma Institute of Pathobiology in an icebox within 3-4 hours of collection. Various patient-related data including age, sex, history of recent antimicrobial usage, marital status, occupation, educational status, hand washing habit, and habit of consumption of raw vegetables and raw meat were collected directly by interviewing the study participants using structured questionnaire to investigate possible factors associated with occurrence of *Salmonella* and antimicrobial resistance.

### 2.3. Isolation, Identification, and Serotyping of *Salmonella*

*Salmonella* isolation was conducted according to Global Foodborne Infections Network, laboratory protocol [17]. Briefly, 1 g of stool sample was suspended in 9 ml of buffered peptone water (Himedia, India) and incubated for 24 hours at 37°C. Then, 100 *μ*l of the suspension was transferred to 10 ml of Rappaport Vassiliadis enrichment broth (RVB), (Oxoid, UK) and incubated for 24 h at 42°C. Suspension of 1 ml of each sample was also transferred to 9 ml of Muller-Kauffmann-Tetrathionate broth (Himedia, India) and Selanite-F broth (Becton-Dickinson, USA) and incubated for 24 h at 37°C. Samples from these three enrichment broths were streaked on to Xylose-Lysine Deoxycholate Agar (Himedia, India), and the plates were incubated at 37°C for 24-48 hours.

Biochemical tests were conducted for presumptive *Salmonella* colonies using Urea, Triple Sugar Iron Agar, Lysine Iron Agar, and Citrate as described elsewhere. Typical *Salmonella* colonies were confirmed using genus specific PCR as described previously [18]. Serotyping of *Salmonella* isolates was conducted at the National Microbiology Laboratory, Office International des Epizooties (OIE) *Salmonella* Reference Laboratory, Public Health Agency of Canada. Somatic (O) antigens were determined using slide agglutination tests whereas flagellar antigens using microplate agglutination technique [19, 20].

### 2.4. Antimicrobial Susceptibility Testing

*Salmonella* isolates were investigated for susceptibility to antimicrobials commonly prescribed for treatment of *Salmonella* or other related enteric pathogens using the Kirby-Bauer disk diffusion method according to Clinical Laboratory Standards Institute guideline [21]. The following antimicrobials (Sensi-Discs, Becton, Dickinson and Company, Loveton, USA) and disc potencies (*μ*g) were used with different concentration, amikacin (30 *μ*g), amoxicillin+clavulanic acid (20/10 *μ*g), ampicillin (10 *μ*g), ceftriaxone (30 *μ*g), cephalothin (30 *μ*g), chloramphenicol (30 *μ*g), ciprofloxacin (5 *μ*g), gentamicin (10 *μ*g), streptomycin (10 *μ*g), sulfisoxazole (1000 *μ*g), sulfamethoxazole+trimethoprim (23.75/1.25 *μ*g), and tetracycline (30 *μ*g). *Escherichia coli* ATCC 25922 was used for a quality control.

### 2.5. Ethical Consideration

The conduct of the study was approved for its ethical issues by the Institutional Review Board of Aklilu Lemma Institute of Pathobiology, Addis Ababa University (Minutes Ref No.: *ALIPB/IRB/014/2011/19*). Prior to sample collection, verbal consent was obtained from the study participants. Study participants were informed about the overall objective of the study, the voluntary participation, and their rights to withdraw from the study if they want to do so.

### 2.6. Data Analysis

Summarized findings were presented in Tables. The association between background characteristics of study participants and occurrence of *Salmonella* was assessed using exact test because some cell frequencies were small violating the assumptions required by Pearson-chi-square test. Associations were reported as being statistically significant whenever *p* value was less than 0.05. Statistical Package for Social Sciences (SPSS, version 20.0) was used to facilitate analysis of the data.

## 3. Results

### 3.1. Characteristics of Study Participants

Out of 298 study participants, 59% were male, 62.3% were married, 41.9% were college/university graduates or studying, and 24.9% were business persons (Table 1).

### 3.2. Prevalence of *Salmonella*

The prevalence of *Salmonella* was 4.0% among males and 5.7% among female diarrheic patients. The isolation of *Salmonella* was highest (8.4%) in the diarrheic patients of age range of 31-45 years although the difference was not statistically significant compared to other age group (*p* value = 0.3). *Salmonella* was detected in 5% (9/178) of those who have the habit of consuming raw vegetables and in 5.7% (5/87) of those who do not have the habit of consuming raw vegetables (Table 2).

### 3.3. Behavior of Study Participants on the Use of Antimicrobials

Out of 241 diarrheic patients involved in this study, 9 (3.7%) reported sharing prescribed antimicrobials with someone else, 181 (78.4%) participants were unaware about antimicrobial resistance, 76 (31.5%) did not complete prescribed dose in a single session of therapy, and 110 (41.5%) participants reported that they get antimicrobials from pharmacy without prescription for various ailments (Table 3).

### 3.4. Serovar Distribution and Antimicrobial Susceptibility of *Salmonella* Isolated from Diarrheic Patients

Four different *Salmonella* serovars were detected in the current study. *Salmonella* Kiambu (*n* = 7) was the most frequently isolated followed by *S*. Saintpaul (*n* = 4), *S*. Haifa (*n* = 2), and *S*. Enteritidis (*n* = 1). The findings of antimicrobial susceptibility test showed that 3 isolates (21.4%) were resistant to sulfisoxazole, 3 isolates (21.4%) were resistant to tetracycline, and 2 isolates (14.3%) were resistant to ampicillin. Three out of 7 *S*. Kiambu isolates (42.9%) were resistant to sulfisoxazole. Resistance to tetracycline was recorded in the three serovars except *S*. Enteritidis. All *Salmonella* isolates were susceptible to amikacin, cephalothin, amoxicillin+clavulanic acid, chloramphenicol, ceftriaxone, ciprofloxacin, gentamicin, streptomycin, and sulfamethoxazole+trimethoprim (Table 4).

Coresistance to ampicillin and tetracycline was detected only in 14.3% (2/14) of the isolates, one from *S*. Kiambu and the other from *S*. Saintpaul. Majority of the isolates were susceptible to most of the antimicrobials investigated, and MDR to over two antimicrobials was not detected.

## 4. Discussion

In the current study, prevalence of *Salmonella* among diarrheic patients was 4.7% which is lower than what was reported in a previous study (7.2%) in Addis Ababa among patients attending government-owned health centers but higher than 1.1% reported from a study in Gondar, North Ethiopia [13] and diarrheic children in Ambo town (1.2%) [22]. Prevalence of *Salmonella* in the current study is in close agreement with previous reports in Addis Ababa (4.5%) [23] and 5.4% prevalence among HIV positive and 4.5% among HIV-negative diarrheic patients in Dessie town, North Ethiopia [24]. Meta-analysis of works done in Ethiopia showed 5.7% prevalence of *Salmonella* in diarrheic adult patients [25]. The difference in isolation rate of *Salmonella* might be explained by difference in socioeconomic status of the diarrheic patients which could be expressed through difference in life style like hand washing and other hygienic practices. Since health services in private hospitals are expensive in Addis Ababa as compared to government hospitals, better ability to pay might be main driving force to attend private hospitals. Most previous studies in Ethiopia recruited study participants from outpatient clinics of government-owned health centers [24–27]. It is believed that only patients with good income can afford to visit private hospitals. In addition, season of sample collection, geographic location, and habit of consuming raw vegetables and raw animal products and illness due to other enteric pathogens could affect the rate of detection of *Salmonella* from diarrheic patients.

In the current study, none of the patient-related factors were shown to have statistically significant association with detection of *Salmonella* from stool of diarrheic patients unlike previous studies where consumption of raw vegetables and stool being watery [26], and poor hand washing practices and consumption of raw/uncooked foods were associated with detection of *Salmonella* in diarrheic patients [28]. The reason why we did not observe such association in this study could be due to small sample size and better practices of cleaning raw vegetables before consumption among study participants.

The most prevalent *Salmonella* serovar in the current study was *S*. Kiambu followed by *S*. Saintpaul. In the previous studies conducted in Addis Ababa, *S*. Typhimurium was the dominant serotype followed by *S*. Virchow [26] whereas these serovars were not isolated in the current study. *Salmonella* Concord and *S*. Typhimurium were reported to be the dominant serovars in diarrheic patients in Addis Ababa in other previous studies [27, 29]. Previous studies in other sub-Saharan African countries also showed dominance of *S*. Typhimurium [30, 31]. Different serovars of *Salmonella* are known to be commonly detected in a given area at different times depending on the type of serovars of *Salmonella* circulating in food animals and food sources in a study area. Epidemiology of NTS is shown to be characterized by temporal variation in dominance of certain *Salmonella* serovars at a time followed by replacement with other serovars [32].

The present study revealed that irrational use of antimicrobials such as failure to finish the whole course of antimicrobials, taking antimicrobials without prescription, and sharing antimicrobials with relatives and or friends were the commonly observed behavior of the study participants. This finding agrees with previous studies conducted in Ethiopia [33] and elsewhere [34]. Such irrational use of antimicrobials, particularly purchase of antimicrobials without prescription, low level of finishing prescribed antimicrobials, and low level of awareness about antimicrobial resistance contributes to emergence and spread of antimicrobial resistance [35]. Hence, special attention should be paid to increase awareness of the community on rational use of antimicrobials.

Relatively low proportions of the *Salmonella* isolates were resistant to sulfisoxazole, tetracycline and ampicillin in this study. Previous study in Ethiopia also showed similar low rate of resistance to tetracycline (13.4%) and ampicillin (14.3%) whereas high rate of resistance to sulfisoxazole (38.9%) was reported [26]. The finding of the current study agrees with previous studies from Greece [36], Libya [37], and England [38]. The current finding is however very low compared to previous reports in Ethiopia [39, 40] where high rate of resistance was recorded in isolates from diarrheic patients and food handlers, respectively. Similarly, unlike previous studies conducted among diarrheic patients in Addis Ababa that reported 3% resistance to ceftriaxone (third-generation cephalosporin) and 4.5% to ciprofloxacin [26], resistance to these antimicrobials was not detected among *Salmonella* isolates tested in the current study. This previous study reported MDR to 5 or more antimicrobials in 25.4% of the isolates. This could be either due to low sample size or relatively better understanding of the study population in the current study on rational use of antimicrobials and may not be exposed to frequent empirical antimicrobial therapy. Moreover, strains of *Salmonella* isolated from patients in the current study might have originated from reservoir hosts or food sources where there is minimal exposure to antimicrobials. Trend analysis of antimicrobial resistance among *S.* Typhimurium isolates from animal meat and humans in China for 20 years showed fluctuation over time [41]. The limitation of this study is low number of samples investigated and the number of samples collected was not equally distributed across each hospital due to low number of diarrheic patients coming to some of the hospitals during the study period.

## 5. Conclusion

The current study showed low prevalence of *Salmonella* in diarrheic patients attending private hospitals in Addis Ababa. Although MDR to several antimicrobials was not detected in the isolates, the observed resistance to some of antimicrobials suggests the need for prudent use of antimicrobials to protect the long-term use of the available antimicrobials.

## Figures and Tables

**Table 1:** Background information of diarrheic patients participated in the study.

Characteristics	Category	Number	Percentage
Sex (*N* = 298)	Male	176	59
	Female	122	40.9

Age group (*N* = 265)	0-5	24	9.1
	6-18	17	6.4
	19-30	76	28.7
	31-45	95	35.8
	>45	53	20

Education (*N* = 265)	Illiterate	20	7.5
	Underage/preschool	24	9
	Primary school (1-8)	36	13.6
	Secondary school (9-12)	74	27.9
	College/university	111	41.9

Marital status (*N* = 265)	Single	100	37.7
	Married	165	62.3

No. of children (*N* = 265)	1-3	100	37.7
	4-6	37	14
	>6	10	3.8
	None	118	44.5

Occupation (*N* = 265)	Businessmen/women	66	24.9
	Student/underage	56	21.1
	Governmental	55	20.8
	Private	44	16.6
	Housewife	34	12.8
	Other	10	3.8

Place of sample collection (*N* = 298)	Bethezatha Hospital	59	19.8
	Ethio Tebib Hospital	59	19.8
	iCMC Hospital	15	5
	Migbaresenay Hospital	8	2.7
	Teklehaimanot Hospital	157	52.7

**Table 2:** Prevalence of *Salmonella* among diarrheic patients attending private hospitals stratified by selected background characteristics.

Characteristics	Category	*Salmonella* status			*p* value
		No. examined	No. positive	Percent	
Sex (*N* = 298)	Male	176	7	4.0	0.6
	Female	122	7	5.7	

Age group (*N* = 265)	0-5	24	1	4.2	0.3
	6-18	17	1	5.9	
	19-30	76	1	1.3	
	31-45	95	8	8.4	
	>45	53	3	5.7	

Educational status (*N* = 265)	Illiterate	16	1	6.3	0.4
	Underage/preschool	25	3	12.0	
	Primary school (1-8)	40	3	7.5	
	Secondary school (9-12)	74	3	4.0	
	College/university	110	4	3.6	

Occupation (*N* = 265)	Business person	66	4	6.0	
	Student/underage	56	2	3.6	
	Governmental	55	3	5.5	0.6
	Private	44	1	2.3	
	Housewife	34	4	11.8	
	Others	10	0	0.0	

^∗^handwashing habit after using toilet (*N* = 241)	Yes	213	10	4.7	0.2
	No	28	3	10.7	

Consumption of raw vegetables (*N* = 265)	Yes	178	9	5.0	0.8
	No	87	5	5.7	

Consumption of raw meat (*N* = 265)	Yes	175	12	6.9	0.2
	No	90	2	2.2	

Stool consistency (*N* = 298)	Loose	168	10	6.0	0.4
	Watery	82	2	2.4	
	Mucoid	36	1	2.8	
	Bloody	12	1	8.3	

Total		298	14	4.7	

^∗^One of the *Salmonella* positive study participants did not respond to this question.

**Table 3:** Antimicrobial usage behavior and practices of study participants.

Characteristics/question of interest	Response category	Number	Percentage
Sharing prescribed antimicrobial with someone and vice versa (*N* = 241)	Yes	9	3.7

Aware about antimicrobial resistance (*N* = 241)	Yes	52	21.6

From where do you usually get antimicrobial drugs (*N* = 265)	From pharmacy with physician prescription	147	55.5
	Without prescription from pharmacy	110	41.5
	With/without prescription	8	3.0

Do you always finish prescribed antimicrobials (*N* = 241)	Yes	165	68.5

**Table 4:** Antimicrobial susceptibility profile of *Salmonella* serovars isolated from stool of diarrheic patients.

Antimicrobials tested	*S*. Enteritidis (*n* = 1)	*S*. Haifa (*n* = 2)	*S*. Kiambu (*n* = 7)	*S*. Saintpaul (*n* = 4)	Total No. (%) of resistant
	No. (%) of resistant	No. (%) of resistant	No. (%) of resistant	No. (%) of resistant	
Amoxicillin+clavulanic acid	0 (0)	0 (0)	0 (0)	0 (0)	0 (0)
Ampicillin	0 (0)	0 (0)	1 (14.3)	1 (25.0)	2 (14.3)
Amikacin	0 (0)	0 (0)	0 (0)	0 (0)	0 (0)
Chloramphenicol	0 (0)	0 (0)	0 (0)	0 (0)	0 (0)
Cephalothin	0 (0)	0 (0)	0 (0)	0 (0)	0 (0)
Ciprofloxacin	0 (0)	0 (0)	0 (0)	0 (0)	0 (0)
Ceftriaxone	0 (0)	0 (0)	0 (0)	0 (0)	0 (0)
Sulfisoxazole	0 (0)	0 (0)	3 (42.9)	0 (0)	3 (21.4)
Gentamicin	0 (0)	0 (0)	0 (0)	0 (0)	0 (0)
Streptomycin	0 (0)	0 (0)	0 (0)	0 (0)	0 (0)
Sulfamethoxazole+trimethoprim	0 (0)	0 (0)	0 (0)	0 (0)	0 (0)
Tetracycline	0 (0)	1 (50.0)	1 (14.3)	1 (25.00)	3 (21.4)

## Data Availability

All data generated or analyzed during this study are included in this published article.

## Abbreviations

AMR: Antimicrobial resistance
DALYS: Disability adjusted life years
MDR: Multidrug resistance
NTS: Nontyphoidal Salmonella
OIE: Office International des Epizooties.
